# DERL3影响肺腺癌细胞A549增殖、侵袭转移的机制

**DOI:** 10.3779/j.issn.1009-3419.2020.104.22

**Published:** 2020-08-20

**Authors:** 丹丹 周, 杰敏 王, 科 杨, 丽萍 张, 荃 郑, 俊 白, 雅琼 胡, 青杰 牟, 崇高 尹, 洪利 李

**Affiliations:** 1 261053 潍坊, 潍坊医学院病理学教研室 Department of Pathology, Weifang Medical University, Weifang 261053, China; 2 261053 潍坊, 潍坊医学院护理学院 Colloge of Nursing, Weifang Medical University, Weifang 261053, China; 3 261053 潍坊, 潍坊医学院临床医学院 College of Clinical Medicine, Weifang Medical University, Weifang 261053, China; 4 261053 潍坊, 潍坊医学院医学研究实验中心 Medical Research Experimental Center, Weifang Medical University, Weifang 261053, China

**Keywords:** 肺肿瘤, 上皮-间质转化, 增殖, 侵袭, 转移, Lung neoplasms, Epithelial-mesenchymal transformation, Proliferation, Migration, Invasion

## Abstract

**背景与目的:**

内质网蛋白降解3(Derlin 3, DERL3)的表达水平与结直肠癌患者的淋巴转移与远处转移密切相关, 然而其在肺腺癌中的表达和生物学行为研究甚少。本研究旨在探讨DERL3在肺腺癌组织中的异位表达及其对肺腺癌A549细胞系侵袭转移的影响, 以揭示其影响肺腺癌侵袭转移的可能机制。

**方法:**

肺腺癌微阵列基因芯片分析有无淋巴结转移肺腺癌组织各3例。GEDS与*Kaplan-Meier* plot查询DERL3在癌症中表达情况与生存曲线。采用Western blot检测细胞中DERL3表达与质粒转染效率。敲低*DERL3*基因后, 采用Transwell检测肺腺癌细胞穿过小室基底膜的数量。采用5-乙炔基-2′脱氧尿嘧啶核苷(EDU)检测细胞增殖能力。采用Western blot方法检测与上皮间质转化相关的E-钙黏蛋白(E-cadherin)和波形蛋白(Vimentin)的表达情况。

**结果:**

微阵列基因芯片结果显示有淋巴结转移的肺腺癌组织中总共有400个表达下调的mRNAs、1, 314个上调的mRNAs。DERL3在肺腺癌组织中明显上调(*P* < 0.05)。生存曲线结果显示DERL3高表达肺癌患者的预后差(*P* < 0.05)。Western blot结果提示质粒敲除成功。敲低DERL3后细胞增殖、迁移与侵袭能力受到抑制(*P* < 0.05)。敲低DERL3后, Vimentin表达水平下降, E-cadherin表达上升(*P* < 0.05)。

**结论:**

敲低*DERL3*基因可抑制A549细胞系的增殖、侵袭与转移能力。

## Abstract (en)

**Background and objective:**

Derlin 3 (DERL3) is downregulated in colorectal cancer (CRC) samples. Its level is closely linked to lymphatic metastasis or distant metastasis rate in CRC patients. However, its biological behavior in lung adenocarcinoma were rarely reported. The aim of this study is to investigate the ectopic expression of DERL3 in lung adenocarcinoma tissues and its effect on the invasion and metastasis of lung adenocarcinoma A549 cell line to reveal the possible mechanism of invasion and metastasis of lung adenocarcinoma.

**Methods:**

Lung adenocarcinoma microarray gene chip data included 3 cases of lymph node metastasis and 3 cases of lung adenocarcinoma tissue without lymph node metastasis. The GEDS and *Kaplan-Meier* plot queries the survival curve and expression level of DERL3. Western blot was used to detect the expression of DERL3 in lung adenocarcinoma cells. The efficiency of knockdown *DERL3* gene was detected by Western blot assay. Transwell detected the number of cells passing through the basement membrane of the transwell. EDU assay detected cell proliferation ability. Western blot detected the expression of epithelial-mesenchymal transition related proteins E-cadherin and Vimentin.

**Results:**

The microarray gene chip results showed that compared with lung adenocarcinoma tissues without lymph node metastasis, 1, 314 mRNAs in lung adenocarcinoma tissues with lymph node metastasis were up-regulated, 400 mRNAs were down (*P* < 0.05). The expression of DERL3 increased in lung adenocarcinoma (*P* < 0.05). The results of survival curve showed that the lung cancer patients with high expression of DERL3 with poor prognosis (*P* < 0.05). Western blot results indicated that plasmid transfection was successful. Knockdown of DERL3 suppressed the ability of proliferation, invasion and migration in A549 cells (*P* < 0.05). After knockdown of DERL3, the expression level of Vimentin was decreased, while E-cadherin expression increased (*P* < 0.05).

**Conclusion:**

Knockdown of DERL3 inhibited the proliferation, invasion and metastasis of A549 cells.

预后差、发病率逐年增高是肺癌患者死亡的主要原因^[1]^。肺癌患者的5年生存率低于20%, 只有16%的患者在早期被诊断^[2]^, 与其他肺癌亚型相比, 肺腺癌的患病率有所上升^[3]^, 越来越多的不吸烟者和从不吸烟者正在发展成肺腺癌^[4]^。因此迫切需要识别有效的生物标志物, 探索新的治疗靶点。

DERL3属于Derlin家族(DERL1、DERL2和DERL3)的一员, 介导错折叠蛋白的内质网相关降解, 是内质网应激反应之一^[5]^。据报道, 内质网应激反应有助于癌症进展^[6, 7]^。虽然DERL1在非小细胞肺癌^[8]^、乳腺癌^[9]^和结肠癌^[10]^中均有报道过高表达, 但尚未有研究探讨DERL3在肺腺癌进展中的作用。

本文将通过研究DERL3在肺腺癌中的异位表达情况进一步探讨其促进肺腺癌侵袭转移的分子机制, 从而确定DERL3是否是肺腺癌潜在的生物标志物和治疗靶点。

## 1 材料与方法

### 1.1 细胞培养与试剂

正常肺上皮BEAS-2B细胞与肺腺癌A549细胞从ATCC(Manassas, VA, USA)获取。BEAS-2B与A549按照ATCC要求常规培养。E-cadherin(1:500)、Vimentin(1:500)单克隆抗体购自Abcam公司。

### 1.2 细胞转染

将对照质粒Scr及敲低DERL3质粒用Lipofectamine 2000转染入A549细胞。质粒由Invitrogen合成。细胞分为两组：①转染对照质粒的细胞命名为Scr/A549;②转染敲低DERL3的质粒的细胞命名为si-DERL3/A549。

### 1.3 Western blot实验

Western blot参照文献^[11]^转染后细胞培养72 h后提取各组蛋白质, 电泳、转膜、封闭, 一抗、二抗孵育后, 用ECL超敏化学发光剂显影, X线胶片曝光。β-actin作为内参。利用NIH Image J软件(Rockville, MD, USA)对Western blot中条带灰度值定量分析, 3次独立重复实验。

### 1.4 5-乙炔基-2′脱氧尿嘧啶核苷(EDU)增殖实验

将各组细胞中加入与培养液等体积的10 mmol/L EDU工作液, 继续孵育细胞2 h后去除培养液, 并加入多聚甲醛室温固定15 min, PBS洗涤细胞3次。每孔用0.3% Triton X-100通透液, 室温孵育15 min, PBS洗涤细胞3次。每孔加入Click反应液, 室温避光孵育30 min。吸除Click反应液, 用PBS洗涤3次。使用Hoechst 33342进行细胞核染色。

### 1.5 Transwell侵袭与迁移实验

按文献^[12]^处理各组细胞, 细胞悬液添加至上室, 下室加入含20%胎牛血清培养液, 培养24 h后4%多聚甲醛固定, 吉姆萨染色后于显微镜下随机选取5个视野计数, 取平均值作为最终结果。实验独立重复3次。

### 1.6 统计学方法

数据均采用SPSS 17.0软件进行统计学分析。数据用均数±标准差表示。两组计量资料之间的比较采用独立样本*t*检验。*P* < 0.05为差异具有统计学意义。

## 2 结果

### 2.1 肺腺癌基因芯片分析

使用肺腺癌mRNAs基因芯片对3例有淋巴结转移的肺腺癌组织(z2, z3, z4)和3例无淋巴结转移肺腺癌组织(t1, t2, t3)的总RNA进行检测, 筛选条件为|logFC|≥2.0(*P* < 0.05)。结果显示有淋巴结转移肺腺癌组织有400个mRNAs表达下调, 1, 314个mRNAs表达上调。其中, DERL3表达上调明显, logFC=11.514, 687, 3。热图显示部分差异表达的mRNAs(图 1)。

**1:**
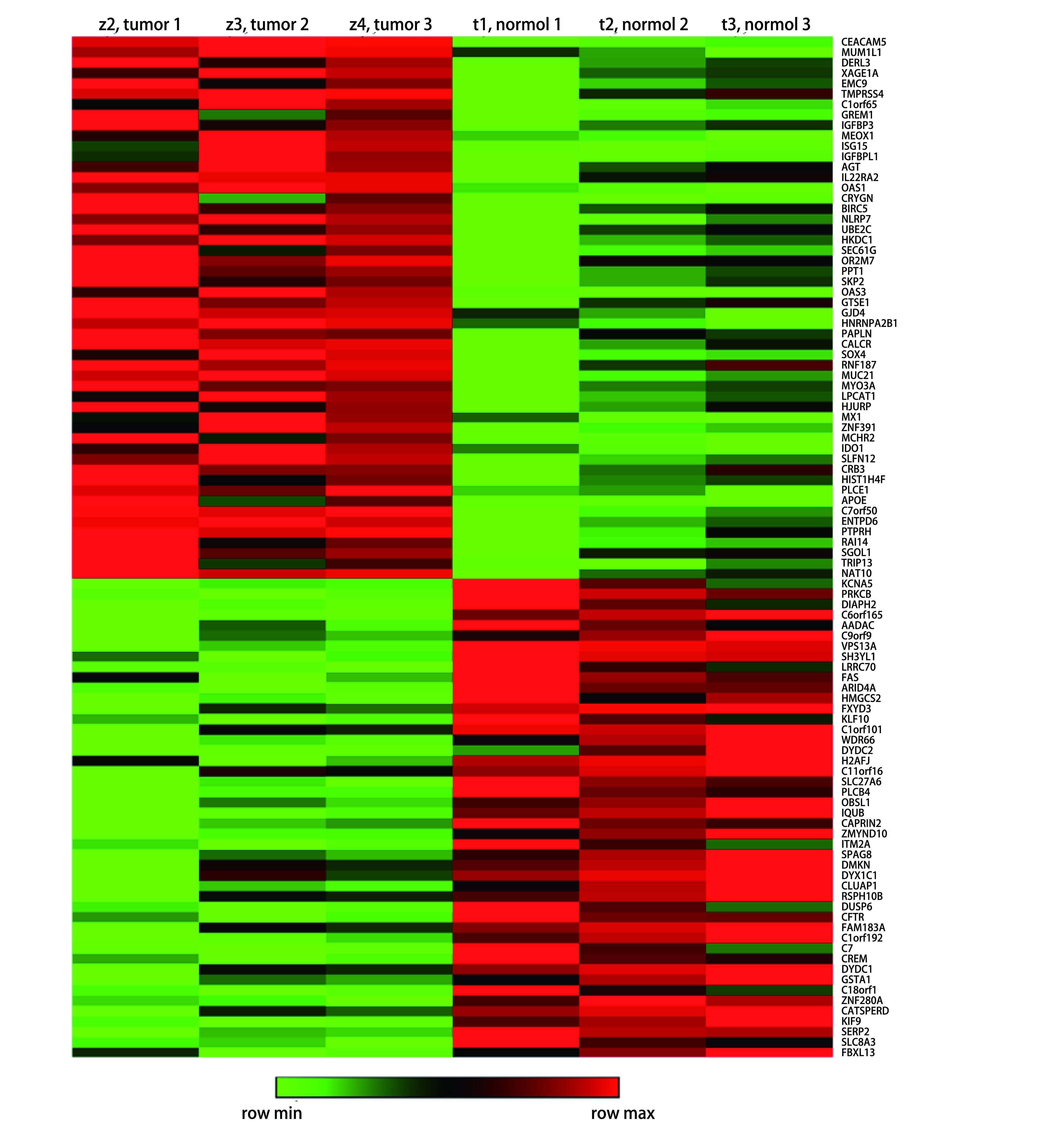
微阵列基因芯片中有或无淋巴结转移部分差异表达mRNA的聚类热图 The cluster heat map of partial differentially expressed mRNAs with or without lymph node metastasis in microarray gene chip

### 2.2 高表达DERL3影响肺癌患者生存预后

使用GEDs在线数据库查询DERL3在癌症中的表达情况, 结果显示DERL3不仅在肺腺癌中表达升高, 还在肺鳞癌(lung squamous cell carcinoma, LUSC)、胰腺癌(pancreatic adenocarcinoma, PAAD)、宫颈鳞癌与宫颈腺癌(cervical squamous cell carcinoma and endocervical adenocarcinoma, CESC)、乳腺浸润癌(breast invasive carcinoma, BRCA)、子宫体子宫内膜癌(uterine corpus endometrial carcinoma, UCEC)、头颈部鳞状细胞癌(head and neck squamous cell carcinoma, HNSC)、膀胱尿路上皮癌(bladder urothelial carcinoma, BLCA)、胆管癌(cholangio carcinoma, CHOL)、肝细胞癌(liver hepatocellular carcinoma, LIHC)、嗜铬细胞瘤和副神经节瘤(pheochromocytoma and paraganglioma, PCPG)、肾透明细胞癌(kidney renal clear cell carcinoma, KIRC)、肉瘤(sarcoma, SARC)、前列腺腺癌(prostate adenocarcinoma, PRAD)、多形性胶质母细胞瘤(glioblastoma multiforme, GBM)中表达升高(图 2A), 在淋巴肿瘤弥漫性大B细胞淋巴瘤(lymphoid neoplasm diffuse large B-cell lymphoma, DLBC)、急性髓性白血病(acute myeloid leukemia, LAML)、卵巢浆液性囊腺癌(ovarian serous cystadenocarcinoma, OV)、胃腺癌(stomach adenocarcinoma, STAD)、睾丸生殖细胞瘤(testicular germ cell tumors, TGCT)、甲状腺癌(thyroid carcinoma, THCA)、结肠腺癌(colon adenocarcinoma, COAD)、直肠腺癌(rectum adenocarcinoma, READ)、间皮瘤(mesothelioma, MESO)、胸腺瘤(thymoma, THYM)、食管癌(esophageal carcinoma, ESCA)、皮肤黑色素瘤(skin cutaneous melanoma, SKCM)、子宫癌肉瘤(uterine carcinosarcoma, UCS)、肾乳头状细胞癌(kidney renal papillary cell carcinoma, KIRP)、肾上腺皮质癌(adrenocortical carcinoma, ACC)、肾嫌色细胞(kidney chromophobe, KICH)、葡萄膜黑色素瘤(uveal melanoma, UVM)、脑低级别胶质瘤(brain lower grade glioma, LGG)中无明显差异或表达降低。所以我们选择DERL3为下一步的研究对象^[13]^。使用*Kaplan-Meier* plot^[14]^查询生存曲线, 结果显示, 高表达DERL3患者生存率明显低于低表达肺癌患者, 差异具有统计学意义(*P*=0.047)(图 2B)。结果说明DERL3在多种癌症中高表达且影响肺癌患者生存预后。

**2:**
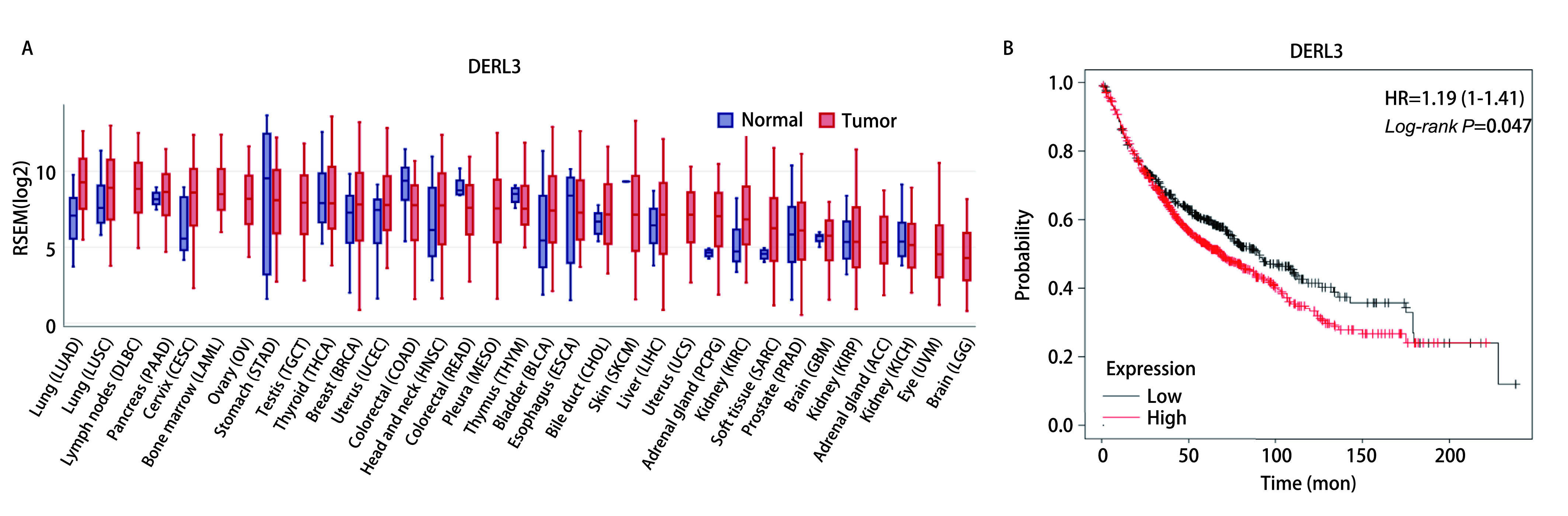
高表达DERL3影响肺癌患者生存预后。A：DERL3在癌组织和癌旁组织的表达；B：DERL3的生存曲线（*P*=0.047）。 High expression of DERL3 affects survival prognosis of lung cancer patients. A: The expression level of DERL3 in tumor and normal adjacent tissues; B: The survive curve of DERL3 in lung cancer patients (*P*=0.047).

### 2.3 DERL3在肺腺癌细胞A549中高表达

使用Western blot实验检测正常肺上皮细胞BEAS-2B和肺腺癌细胞A549中DERL3的蛋白表达程度。结果显示, DERL3在A549中的表达明显高于BEAS-2B, 差异具有统计学意义(*P* < 0.05)(图 3)。

**3:**
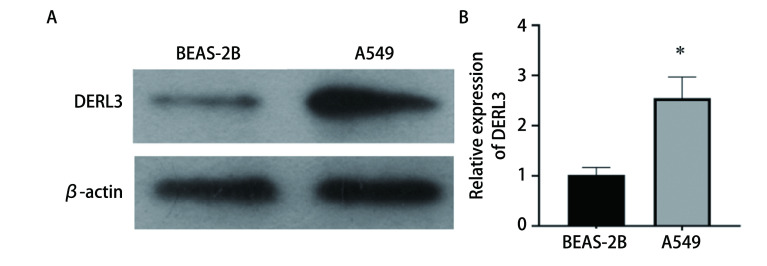
Western blot检测DERL3在肺腺癌A549细胞和正常肺上皮BEAS-2B细胞中的表达。A：Western blot验证DERL3表达; B：相对灰度值表达比较柱状图(^*^P < 0.05)。 The expression of DERL3 in lung adenocarcinoma A549 cells and normal lung epithelial BEAS-2B cells was detected by Western blot. A: Western blot to verify DERL3 expression; B: The bar chart compares the expression of relative gray values (^*^P < 0.05).

### 2.4 si-DERL3质粒敲减效率

Western blot实验检测si-DERL3质粒转染效率。结果显示, 与对照组细胞Scr/A549相比敲减组DERL3表达显著降低, 提示敲减质粒转染成功, 差异具有统计学意义(*P* < 0.01)(图 4)。

**4:**
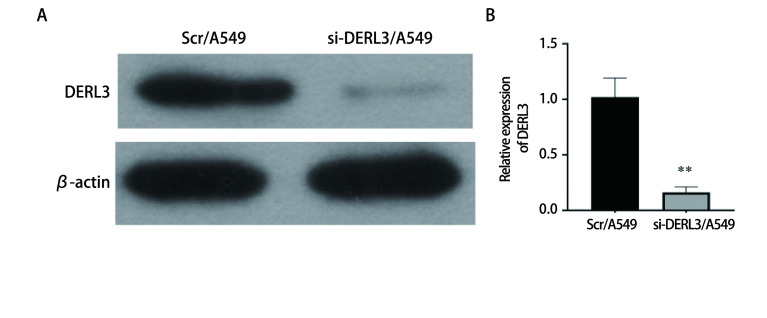
使用Western blot检测DERL3敲减效率。A：Western blot验证DERL3表达; B：相对灰度值表达比较柱状图(^**^P < 0.01)。 The DERL3 knockdown efficiency was detected by Western blot. A: Western blot to verify DERL3 expression; B: The bar chart compares the expression of relative gray values (^**^P < 0.01).

### 2.5 DERL3促进A549细胞的迁移与侵袭能力

Transwell实验测定DERL3对A549细胞迁移与侵袭能力的影响。结果显示, 与Scr/A549组相比, si-DERL3/A549组的细胞迁移与侵袭能力明显降低(迁移：0.594±0.033 *vs* 1.162±0.038;侵袭：0.413±0.069 *vs* 1.113±0.024), 差异具有统计学意义(*P* < 0.05)(图 5)。结果表明DERL3可以提高肺腺癌细胞的迁移与侵袭能力。

**5:**
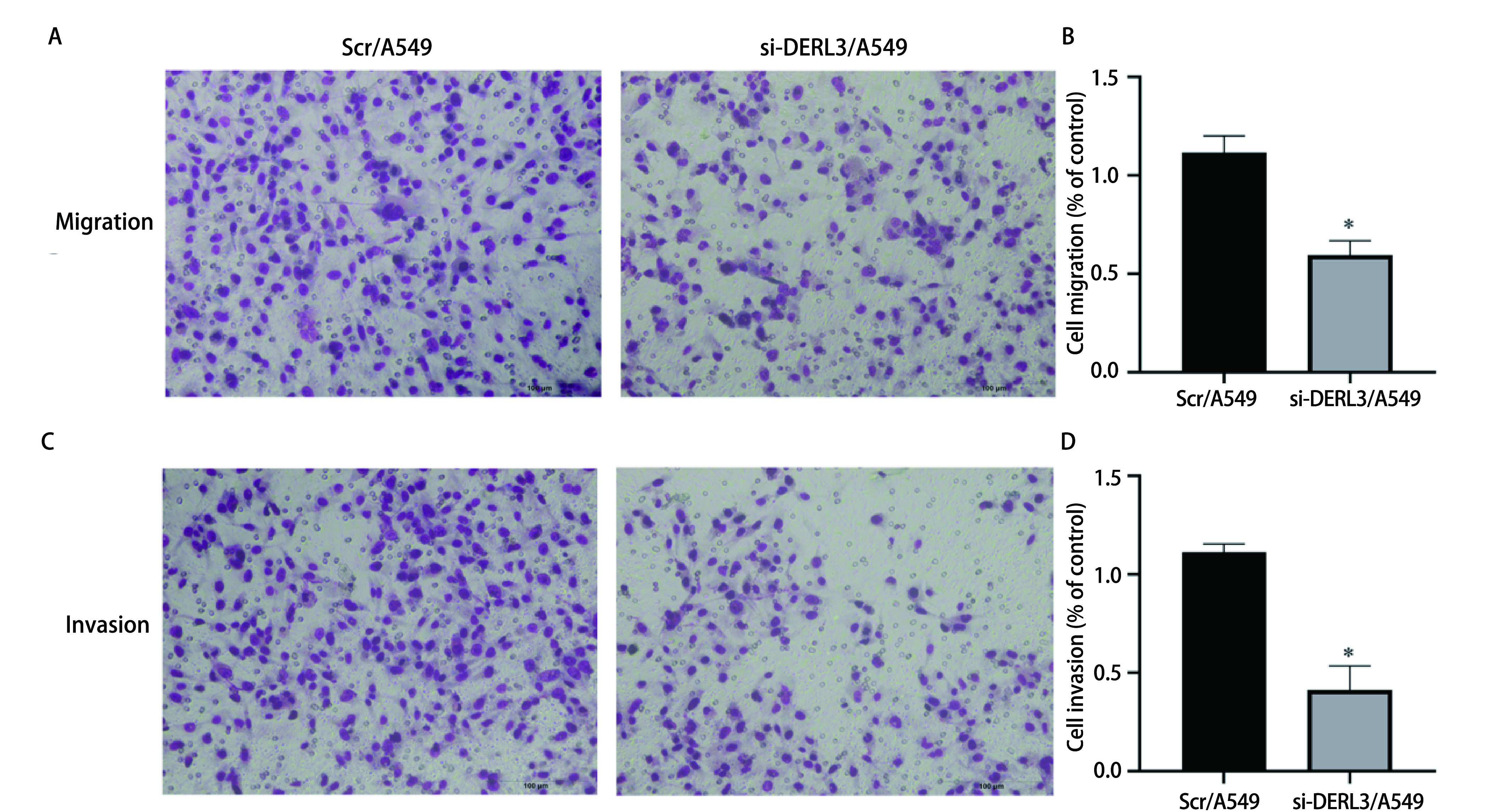
Transwell实验检测转染后肺腺癌细胞迁移与侵袭能力。A：迁移实验检测穿过基底膜细胞数量; B：细胞迁移率的柱状图比较(^*^P < 0.05);C：侵袭实验检测穿过基底膜细胞数量; D：细胞侵袭率的柱状图比较(^*^P < 0.05)。 The ability of invasion and migration of lung adenocarcinoma cell after transfection was detected by transwell. A: The number of cells passing through the basement membrane was detected by the migration experiment; B: Histogram comparison of cell mobility (^*^P < 0.05); C: Invasion assay detected the number of cells passing through basement membrane; D: Histogram comparison of cell invasion rate (^*^P < 0.05).

### 2.6 敲低DERL3抑制肺腺癌细胞增殖能力

EDU实验检测Scr/A549与si-DERL3/A549细胞增殖能力, 结果显示, 与Scr/A549组相比, si-DERL3/A549组EDU染色阳性率明显降低, 表明该组增殖能力受到了明显的抑制, 差异具有统计学意义(*P* < 0.05)(图 6)。

**6:**
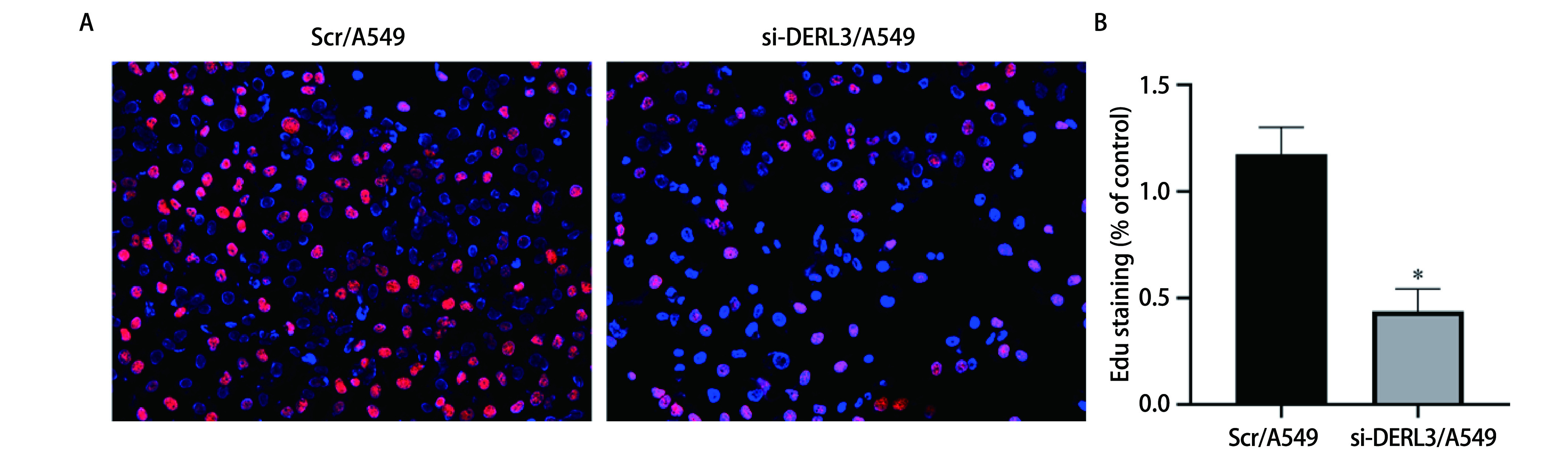
EDU实验检测敲低DERL3后A549细胞增殖能力。A：红色代表EDU染色阳性的细胞, 蓝色代表细胞核; B：EDU染色阳性率的柱状图比较(^*^P < 0.05)。 EDU assay detected the proliferation ability of A549 cells after knockdown of DERL3. A: Red for EDU positive cells, blue for nucleus; B: Histogram comparison of positive rate of EDU staining (^*^P < 0.05).

### 2.7 肺腺癌A549细胞中E-cadherin以及Vimentin的表达水平

Western blot检测Scr/A549与si-DERL3/A549中的E-cadherin以及Vimentin的表达。si-DERL3/A549组中Vimentin下降, E-cadherin的表达上升, 差异具有统计学意义(*P* < 0.05)(图 7)。

**7:**
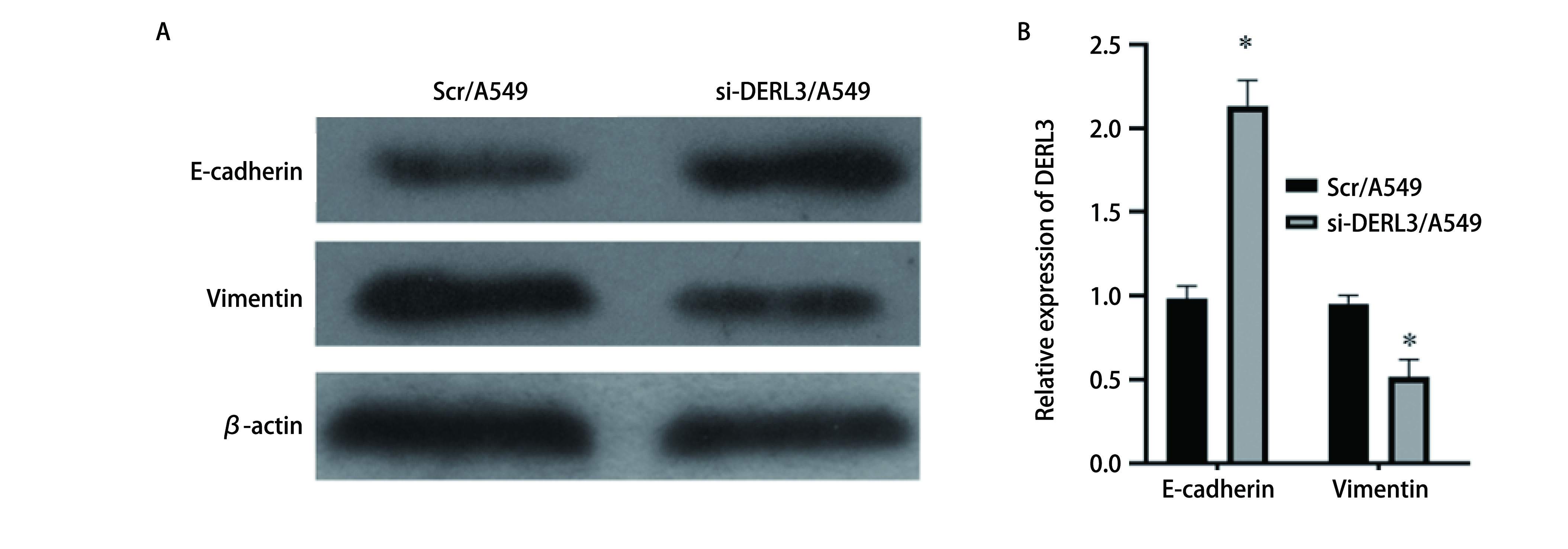
Western blot检测E-cadherin以及Vimentin在Scr/A549和si-DERL3/A549细胞中的表达。A：Western blot验证E-cadherin以及vimentin表达; B：相对灰度值表达比较柱状图(^*^P < 0.05)。 The expression of E-cadherin and Vimentin in Scr/A549和si-DERL3/A549 cells was detected by Western blot. A: Western blot to verify the expression of E-cadherin and vimentin; B: The bar chart compares the expression of relative gray values (^*^P < 0.05).

## 3 讨论

肺癌5年相对生存率只有17%, 是全球癌症死亡的主要缘由之一^[15]^, 因为大部分患者在诊断时已经转移, 或者在初次手术或放疗后复发。本文通过对肺腺癌基因芯片的分析找到在淋巴结转移的肺腺癌组织中表达较高的*DERL3*基因, 使用在线数据库进行预测, 结果发现其在多种癌症中高表达, 且高表达*DERL3*基因的肺癌患者生存率明显低于低表达患者。所以在本研究中我们将关注DERL3在肺腺癌细胞侵袭、转移方面的作用。

已有文献^[16-19]^报道DERL3通过激活PI3K/AKt和EGRF-EPK途径促进癌细胞增殖和侵袭能力, 从而参与肺癌的恶性表型。我们通过在线数据库发现DERL3不仅在肺腺癌中高表达, 在肺鳞癌、胰腺癌、宫颈癌、骨髓瘤、甲状腺癌等多种癌症中表达升高, 这说明DERL3可能作为癌基因调控多种癌症的发展过程。此外, 生存曲线结果表明高表达DERL3肺癌患者预后较差。随后我们通过Transwell迁移和侵袭实验证明敲减DERL3后穿过基底膜细胞数明显降低, 说明敲减DERL3能够抑制细胞迁移与侵袭能力。EDU增殖实验证实敲减DERL3后细胞EDU染色阳性率明显下降, 说明敲减DERL3能够抑制A549细胞增殖能力。以上实验证实DERL3可能通过促进肺腺癌A549细胞增殖、迁移和侵袭能力从而促进肺腺癌发生发展过程。

上皮间质转化是由一个充分分化的上皮细胞转化为间充质表型的过程, 伴有上皮标志物的丢失、间充质特性的获得、迁移增强的细胞骨架的重组^[18]^。上皮间质转化在生理和病理上参与多种癌症的癌变过程^[19]^。已有文献^[16, 17]^报道在非小细胞肺癌和膀胱癌中, DERL1的过表达通过影响上皮-间质转化从而影响癌细胞侵袭转移。而我们证实敲减A549中的DERL3导致E-cadherin上调, Vimentin下调, 所有这些生物标志物变化提示上皮细胞向间充质细胞的转变。DERL3在肺腺癌细胞A549中通过调控上皮间质转化从而导致更恶性的表型。这是关于DERL3在上皮间质转化中作用的第一篇报道。

此外本研究有一定的局限性, 这些结果来自体外数据, 需要通过体内研究来进一步验证。DERL3在其他癌症中的分子机制有赖于进一步探究。综上所述, DERL3能促进肺腺癌恶性表型, 在肺腺癌组织中DERL3的高表达导致预后不良, 可能是一个潜在的肺腺癌预后标志物。
